# Heartbeat detection and personal authentication using a 60 GHz Doppler sensor

**DOI:** 10.3389/fdgth.2025.1570144

**Published:** 2025-08-21

**Authors:** Takuma Asano, Shintaro Izumi, Hiroshi Kawaguchi

**Affiliations:** Architecture Laboratory, Graduate School of Science, Technology and Innovation, Kobe University, Kobe, Japan

**Keywords:** biometric authentication, conditional variational autoencoder (CVAE), heartbeat, microwave Doppler sensor, non-contact measurement

## Abstract

**Background:**

Microwave Doppler sensors, capable of detecting minute physiological movements, enable the measurement of biometric information, such as walking patterns, heart rate, and respiration. Unlike fingerprint and facial recognition systems, they offer authentication without physical contact or privacy concerns. This study focuses on non-contact seismocardiography using microwave Doppler sensors and aims to apply this technology for biometric authentication.

**Method:**

We proposed a method for authenticating and identifying heartbeat signals through supervised learning using a conditional variational autoencoder (CVAE). A 60 GHz microwave Doppler sensor was used to capture heartbeat signals, which were processed using a conformer network to detect peaks and segment individual beats. High signal-to-noise ratio waveforms were selected, and time-frequency analysis extracted relevant features. Spectrograms labeled with subject data were input into the CVAE, which encoded subject-specific features into a latent space for authentication.

**Results:**

The proposed heartbeat-based authentication method, validated on 13 subjects, achieved an average balanced accuracy of 97.3% for authentication and an average accuracy of 94.7% for identification. Compared with conventional methods, this approach demonstrated superior performance by effectively encoding subject-specific features while mitigating noise-related challenges.

**Conclusion:**

The proposed method enhanced the feasibility of non-contact heartbeat-based authentication by achieving high accuracy while addressing noise-related challenges. Its application could improve biometric security without compromising user privacy. Further advancements in handling posture variations and scalability are essential for real-world implementation.

## 1 Introduction

Biometric authentication extracts unique features, such as facial structures and fingerprints, from biometric data to identify individuals. Unlike traditional authentication methods, it does not require users to memorize passwords, enter information, or carry physical identification. Moreover, biometric authentication is more secure because it is difficult to impersonate and less vulnerable to theft. Given these advantages, there is growing demand for new biometric modalities that enhance both usability and security, especially non-contact methods that reduce hygiene risks.

Among various modalities, heart-based biometric authentication has gained attention because it reflects internal physiological characteristics that are difficult to forge and can be measured noninvasively. Individual differences in cardiac muscle thickness, electrical conduction, and vascular elasticity contribute to unique heartbeat patterns (1), and personal identification can be derived by analyzing the amplitude and duration of the P, Q, R, S, and T waves in an electrocardiogram (ECG). Typically, ECGs are recorded by measuring electrical potential differences using electrodes attached to the body surface. Various ECG-based biometric authentication methods have also been proposed. Arteaga-Falconi et al. (2) introduced an ECG-based algorithm that enables authentication by lightly touching a mobile device, pioneering ECG authentication for mobile applications. Choi et al. (3) developed preprocessing and feature extraction techniques to improve authentication robustness against noise in mobile ECG data. Gutta and Cheng (4) proposed a method that simultaneously optimizes feature extraction and classifier design, and Sun et al. (5) utilized individualized autoencoders for personalized ECG authentication.

However, contact-based methods face practical challenges such as the inconvenience of electrode attachment, hygiene concerns, and user discomfort. To address these issues, non-contact techniques have been proposed using microwaves or accelerometer sensors to detect heartbeats or body surface vibrations. Lin et al. (6) proposed a non-contact authentication system utilizing continuous wave radar to sense cardiac motion. Rissacher and Galy (7) used a 2.4 GHz radar system with continuous wavelet transform (CWT) feature extraction, while Okano et al. (8) applied autoregressive (AR) models and spectrogram analysis to heartbeat signals captured by a 24 GHz Doppler sensor. Shi et al. (9) performed personal identification with 24 GHz radar and support vector machines (SVM). Cao et al. (10) extracted micro-Doppler signals using short-time Fourier transform (STFT) and classified them with deep convolutional neural networks (DCNN). Huang et al. (11) achieved continuous authentication based on near-field heart rate signals using an accelerometer sensor, employing a convolutional autoencoder (CAE). Wu et al. (12) combined ultra-wideband (UWB) radar and convolutional neural networks (CNN) for heartbeat-based identification. Hinatsu et al. (13) proposed a biometric authentication method based on measuring subtle displacements of the chest wall using a very high frequency band loop antenna, and Kobayashi et al. (14) introduced a personal identification approach that combines respiratory and heartbeat-derived features measured by a 79 GHz millimeter-wave radar.

**Figure 1:**
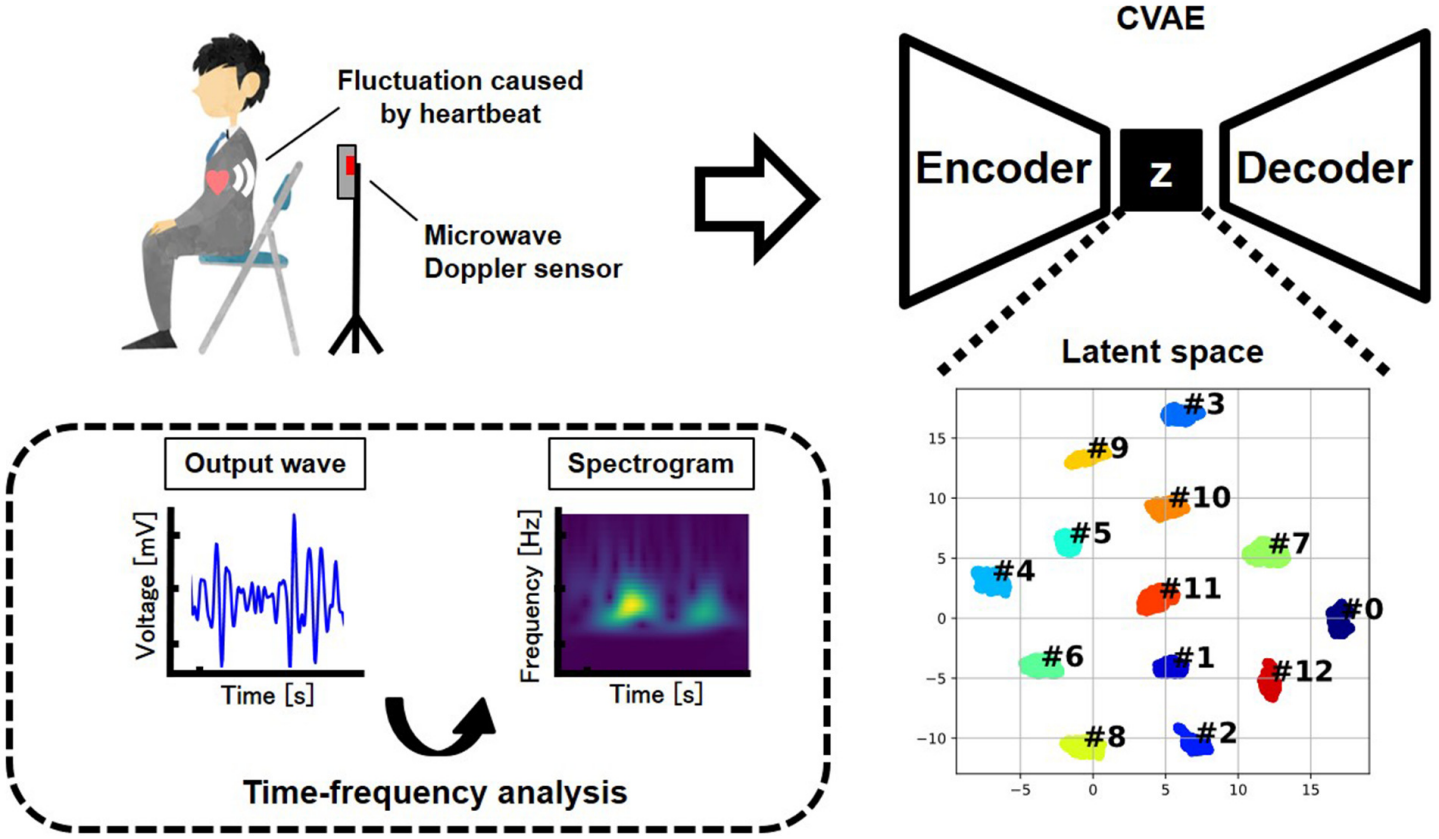
Concept of the proposed authentication method.

In this study, we propose a biometric authentication framework that combines a deep generative model, specifically a conditional variational autoencoder (CVAE) (15, 16), with non-contact heartbeat measurement using a 60 GHz microwave Doppler sensor. By employing a CVAE, the latent feature space is structured based on subject IDs, enabling robust classification even under signal variability. The design concept of the proposed authentication system is illustrated in Figure 1.

## 2 Materials and methods

### 2.1 Microwave Doppler sensor

The time intervals of small displacements on the body surface due to heartbeats strongly correlate with the interbeat intervals in an ECG. Therefore, heartbeats can be detected by measuring these body surface displacements without directly measuring the ECG. In this study, a Doppler sensor utilizing microwaves in the 60 GHz band was used to measure surface displacements.

When microwaves are emitted toward an object, the frequency of the reflected waves shifts based on the object's velocity due to the Doppler effect. The microwave Doppler sensor receives the reflected waves and outputs signals by mixing the transmitted and received waves. If the transmission frequency is $f_{0}[\mathrm{Hz}]$, the velocity of the object irradiated by the microwaves is v[m/s], and the speed of light is c[m/s]. The receiving frequency $f_{r}[\mathrm{Hz}]$ can be expressed using the Doppler effect, as shown in (Equation 1):

(1) $$f_{r}=\frac{c+v}{c-v}f_{0}=f_{0}+\frac{2v}{c-v}f_{0}\approx f_{0}+\frac{2v}{c}f_{0}(∵c\gg v)$$

where the frequency shift of the reflected wave relative to the velocity of the object is proportional to the frequency of the transmitted wave. Therefore, higher transmitted frequencies provide higher resolutions. Vibrations on the body surface induced by heartbeats have displacements of <1 mm. Radio waves at 60 GHz are mostly reflected by the body surface; however, this frequency is sufficient to capture the heartbeat component from the body surface.

A Doppler sensor (BGT60LTR11AIP, Infineon Technologies) was used for this experiment, with a sampling rate set to 250 Hz. The measured data were wirelessly transmitted via Bluetooth low energy (LE) and transferred to a personal computer (PC) in real time.

### 2.2 Measured example of heartbeat

**Figure 2:**
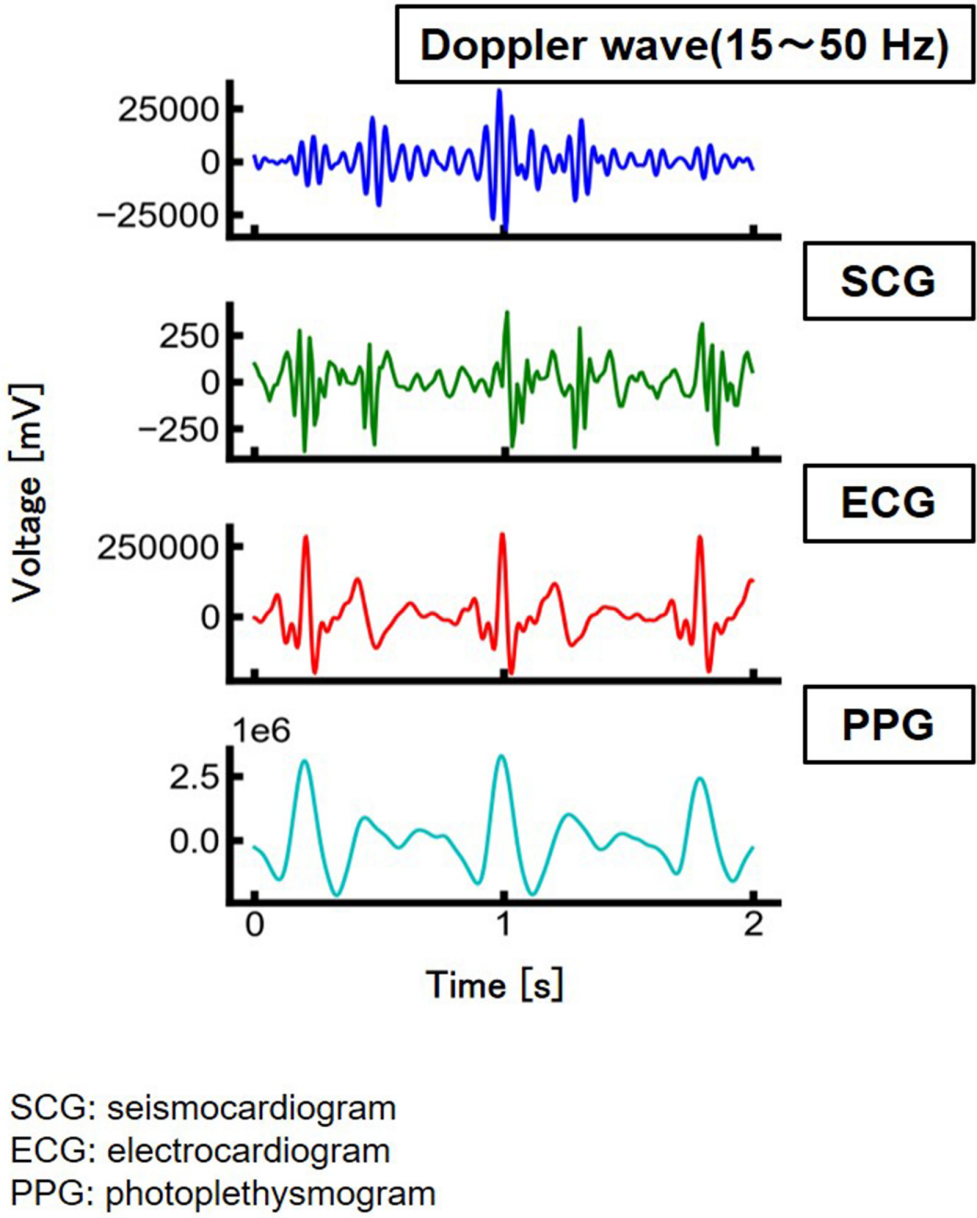
Measurement results of heartbeat.

Figure 2 shows the measurement results obtained from the human body (back) using a microwave Doppler sensor. Reference data were measured using a patch-type ECG sensor (SEN0213, Zhiwei Robotics Corp.) attached to both hands and the left leg. The body surface oscillations corresponding to systole and diastole were also measured (Figure 2). The waveform obtained using the microwave Doppler sensor was similar to that obtained using the seismocardiogram (SCG) (17), supporting the hypothesis that a microwave Doppler sensor can measure the SCG without physical contact.

### 2.3 Measurement and dataset construction

**Figure 3:**
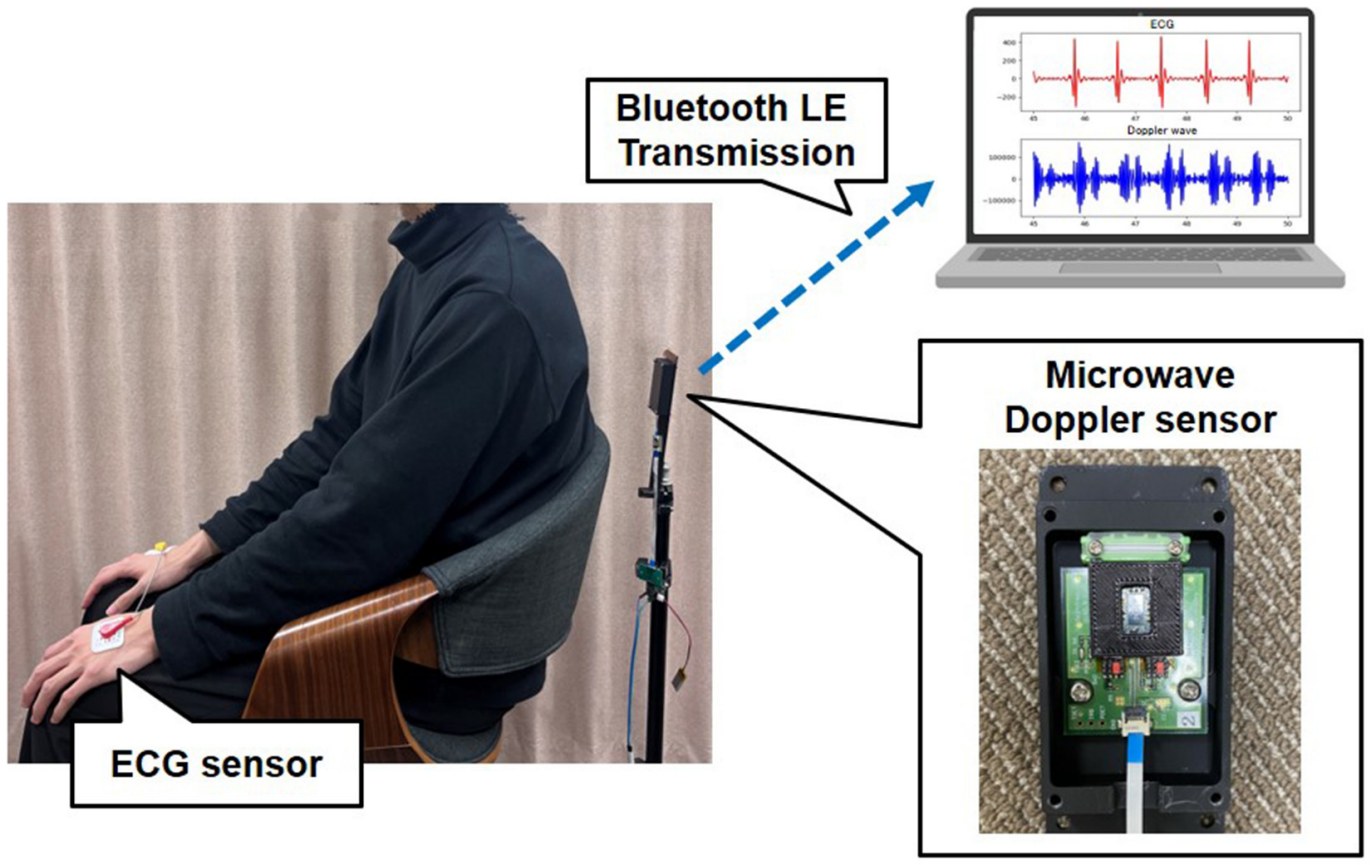
Measurement conditions.

Thirteen subjects (11 males and 2 females, aged 22–24 years) were measured to evaluate the performance of the proposed method under the conditions shown in Figure 3. The measurement duration for each subject was 60 s. The heartbeat was measured while the subjects sat and breathed normally. The measured data were segmented using the method described later, retaining only data with a high signal-to-noise ratio. The frequency region containing the heartbeat component was extracted using a time-frequency analysis. Furthermore, a five-beat time-series moving average was then calculated, followed by data augmentation. This study was approved by the Ethics Committee of the Graduate School of System Informatics, Kobe University (approval number: R03-02). All procedures adhered to the ethical standards of the Institutional and National Research Committee and the 1964 Helsinki Declaration, along with its later amendments or comparable ethical standards.

### 2.4 Preprocessing

The measured time series of the Doppler waves was used to create a dataset for machine learning.

The time-series waveform obtained by the microwave Doppler sensor included various types of noise, such as environmental noise (including ambient and body movement noise), internal noise from the sensor circuit, external noise affecting the sensor. To reduce the impact of noise and respiration, a bandpass filter was applied to allow only specific frequency bands to pass. In this study, a bandpass filter with a passband of 15–50 Hz was applied. This frequency range was carefully selected because, when using a 60 GHz Doppler sensor, heartbeat-induced micro-vibrations are primarily observed within the 15–50 Hz range, whereas respiratory movements mainly appear at frequencies below 10 Hz. Setting the lower cutoff frequency at 15 Hz effectively suppresses respiration components while preserving heartbeat signals. Additionally, the upper cutoff frequency at 50 Hz helps to eliminate high-frequency noise, including 60 Hz power-line interference, thereby improving signal quality for heartbeat detection.

The R-wave peaks of the heartbeats were then extracted from the Doppler waves using a machine learning approach. In this study, we utilized the conformer (18) model to estimate the R-wave peaks of heartbeats. Specifically, Doppler waves measured separately from those used for authentication were used as input features, and the R-wave peaks of the ECG, processed with a 1 Hz high-pass filter, served as the ground truth for training. The trained model was then used to estimate the R-wave peaks from the Doppler waves measured for authentication. Ground-truth data were prepared by assigning a value of one to the R-wave peaks and a value of zero to all the other points. The data were then converted into a triangular waveform format. The conformer is a machine learning model that combines CNN and transformer architectures, primarily used in natural language processing. For the heartbeat peak extraction task using the conformer, data from 13 subjects, with 60 s of data per subject, were used for training. Overlapping processing was applied to compensate for the limited data. The machine-learning model consisted of five conformer blocks with dimensions of 30. Each block included a multihead self-attention module with three attention heads, a convolution module with a kernel size of 31, and a feed-forward module with 512 dimensions. The batch size was set to 512, the number of epochs to 300, the loss function to mean squared error (MSE), the optimizer to Adam, and the learning rate to 0.0001.

**Figure 4:**
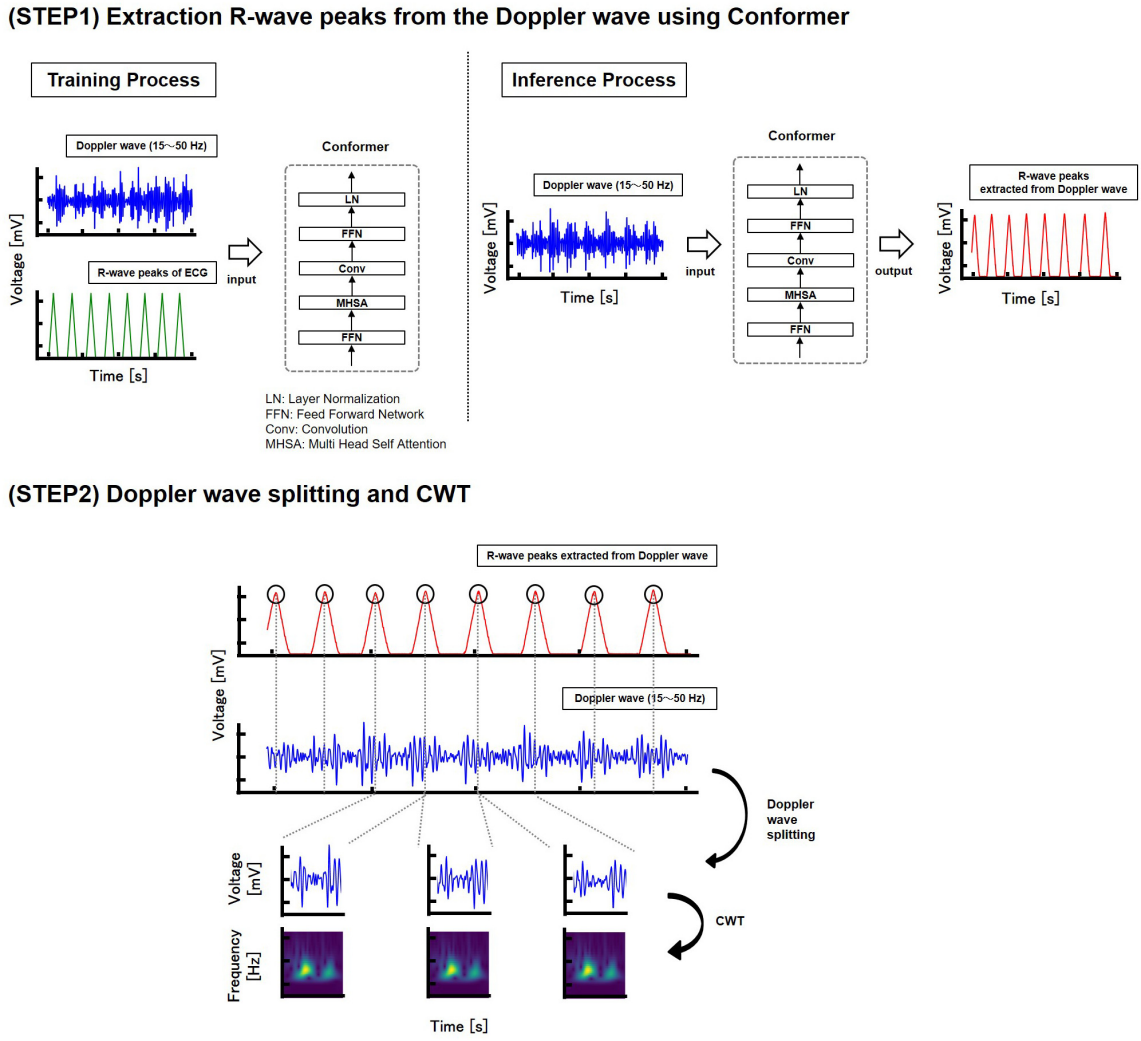
Dataset construction.

After downsampling the waveform of the extracted peak and Doppler wave to 125 Hz, the Doppler wave was segmented based on the peak. The signal-to-noise ratio was then calculated for each segmented Doppler waveform, selecting only those with high signal-to-noise ratios. In this study, the signal-to-noise ratio was calculated using the peak value as the signal and the average value of the Doppler waveform as the noise. Doppler waveforms with a signal-to-noise ratio of four or higher were used for the training data, while those with a signal-to-noise ratio of six or higher were used for the test data. The signal-to-noise ratio was set slightly lower for the training data than for the test data to enhance the data robustness by adding noise. Subsequently, a time-frequency analysis was conducted using a CWT (see Figure 4).

The challenge in non-contact measurements is the isolation of body motion noise. Doppler waves contain noise from body motion and the surrounding environment. However, Doppler shifts due to breathing and body movement are smaller than those caused by heartbeats and can be distinguished using frequency-domain computation. Therefore, we extracted the heartbeat component by using a spectrogram as an input to a neural network.

The resolution of the time-frequency analysis should be considered carefully. The STFT is a widely used method for time-frequency analysis; however, it struggles to achieve both frequency and time resolution owing to the uncertainty principle of the Fourier transform (19). Therefore, time-frequency analysis was conducted using the CWT, as shown in (Equation 2):

(2) $$W(a,b)=|a{|}^{\left(-1/2\right)}\int_{-\infty }^{\infty }x(t)\psi \left(\frac{t-b}{a}\right)dt$$

where x(t) is the signal to be analyzed, ψ is the mother wavelet, a is the scale parameter corresponding to the frequency to be extracted, and *b* is the shift parameter corresponding to the time to be analyzed. In this study, the Morlet wavelet with $\omega_{0}=6$ is used as the mother wavelet (20), as shown in (Equation 3):

(3) $$\psi (t)=\pi^{-\frac{1}{4}}e^{i\omega_{0}t}e^{-\frac{t^{2}}{2}}$$

Finally, data augmentation (21) was applied to the spectrograms. This included time shifts, where data points on the time axis were shifted forward and backward by ±2 points; time stretching, where the length on the time axis was altered; mixup (22), which linearly combined two spectrograms; the addition of white noise, a random signal with equal intensity across all frequencies; and the calculation of a moving average over every five beats.

### 2.5 CVAE model

In this study, we employed a CVAE (15, 16) as the machine learning model for heartbeat-based biometric authentication. The CVAE is a deep generative model proposed by Sohn et al., based on the Variational Autoencoder (VAE) (23), which was originally introduced by Kingma et al.

While the standard VAE learns an unsupervised latent representation of input data, the CVAE extends this framework to support supervised learning by conditioning the latent space on class labels. The VAE encodes input data into latent variables and decodes outputs from compressed representations to reconstruct the original input. In contrast, the CVAE controls the distribution over the latent space based on the labels assigned during training, thereby enabling the model to structure the latent space according to class-specific information.

Let $\left(X,Y)=\{\left(x_{1},\;y_{1}),\;\ldots ,\;(x_{N},y_{N}\right)\right\}$ represent the measured data and their corresponding class labels, where $x_{i}\in R^{D}$ denotes the input data and $y_{i}\in 0,1,2,\;\ldots ,\;K-1$ denotes the subject ID. Each data point $x_{i}$ is associated with a latent variable $\;z_{i}$. The decoder distribution $p_{\theta }(x|z)$ and the prior distribution $p_{\psi }(z|y)$ are defined by (Equations 4 and 5):

(4) $$p_{\theta }(x|z)=\mathrm{Bern}(\mu_{\theta }(z))$$

(5) $$p_{\psi }(z|y)=N(\mu_{\psi }(y),\sigma_{\psi }^{2}(y))$$

θ and ψ are hyperparameters of the neural network in (Equations 4 and 5). The posterior distribution is approximated as in (Equation 6). ϕ is a hyperparameter.

(6) $$q_{\phi }(z|x)=N(\mu_{\phi }(x),\sigma_{\phi }^{2}(x))$$

The training objective is to maximize the conditional log-likelihood $\log p_{\theta ,\psi }(x|y)$, which is intractable and instead approximated by maximizing the variational lower bound. This bound is given by the expected log-likelihood of the reconstructed input minus the Kullback–Leibler (KL) divergence between the approximate posterior and prior distributions, as shown in (Equation 7):

(7) logpθ,ψ(x|y)=>∫qϕ(z|x)logpθ(x,z|y)qϕ(z|x)dz=∫qϕ(z|x)logpθ(x|z)pψ(z|y)qϕ(z|x)dz=Eqϕ(z|x)[logpθ(x|z)]−DKL[qϕ(z|x)||pψ(z|y)]=−L′CVAE(x,y;θ,ϕ,ψ)

The CVAE model used in this study follows the structure proposed by Sohn et al., with a key modification: the encoder $q_{\phi }(z|x,y)$ is simplified to $q_{\phi }(z|x)$, excluding the label *y* from the encoder input. This modification allows the encoder to autonomously extract individual-specific features from the input data without explicitly requiring label information during encoding, while the prior remains label-dependent.

**Figure 5:**
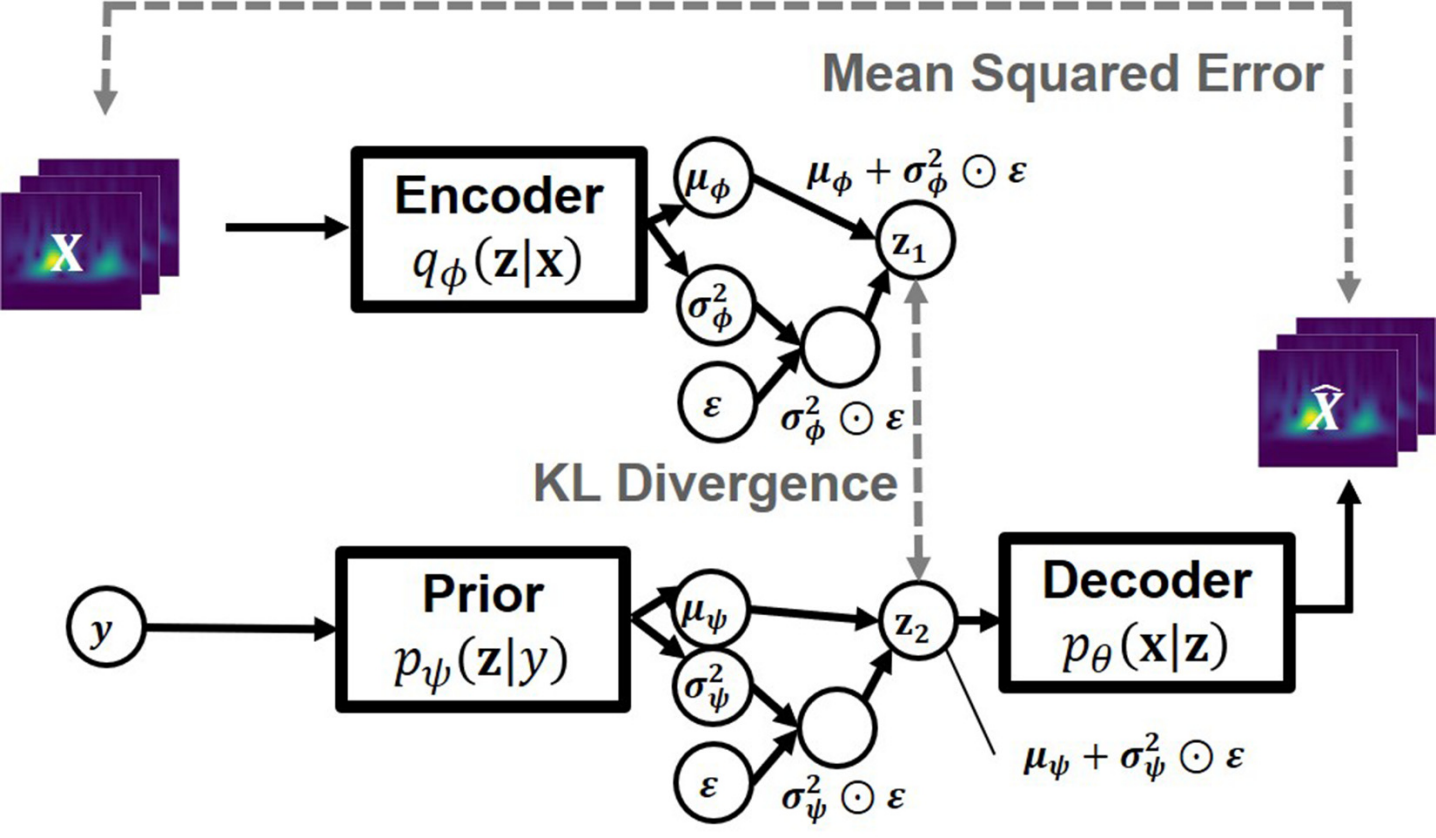
Architecture of the proposed model.

The overall model architecture is illustrated in Figure 5. The encoder network predicts the mean and variance of the latent distribution, from which the latent variable *z* is sampled using the reparameterization trick (23), expressed as $z=\mu +\epsilon ⊙\sigma^{2}$, where ϵ∼N(0,I) follows a standard normal distribution. Simultaneously, the prior network generates a label-conditioned prior distribution. The KL divergence between the encoder's output and the label-conditioned prior is minimized to structure the latent space, while the decoder reconstructs the input from the latent variable. The overall loss function is the sum of the reconstruction loss, computed using MSE, and the KL divergence term.

**Figure 6:**
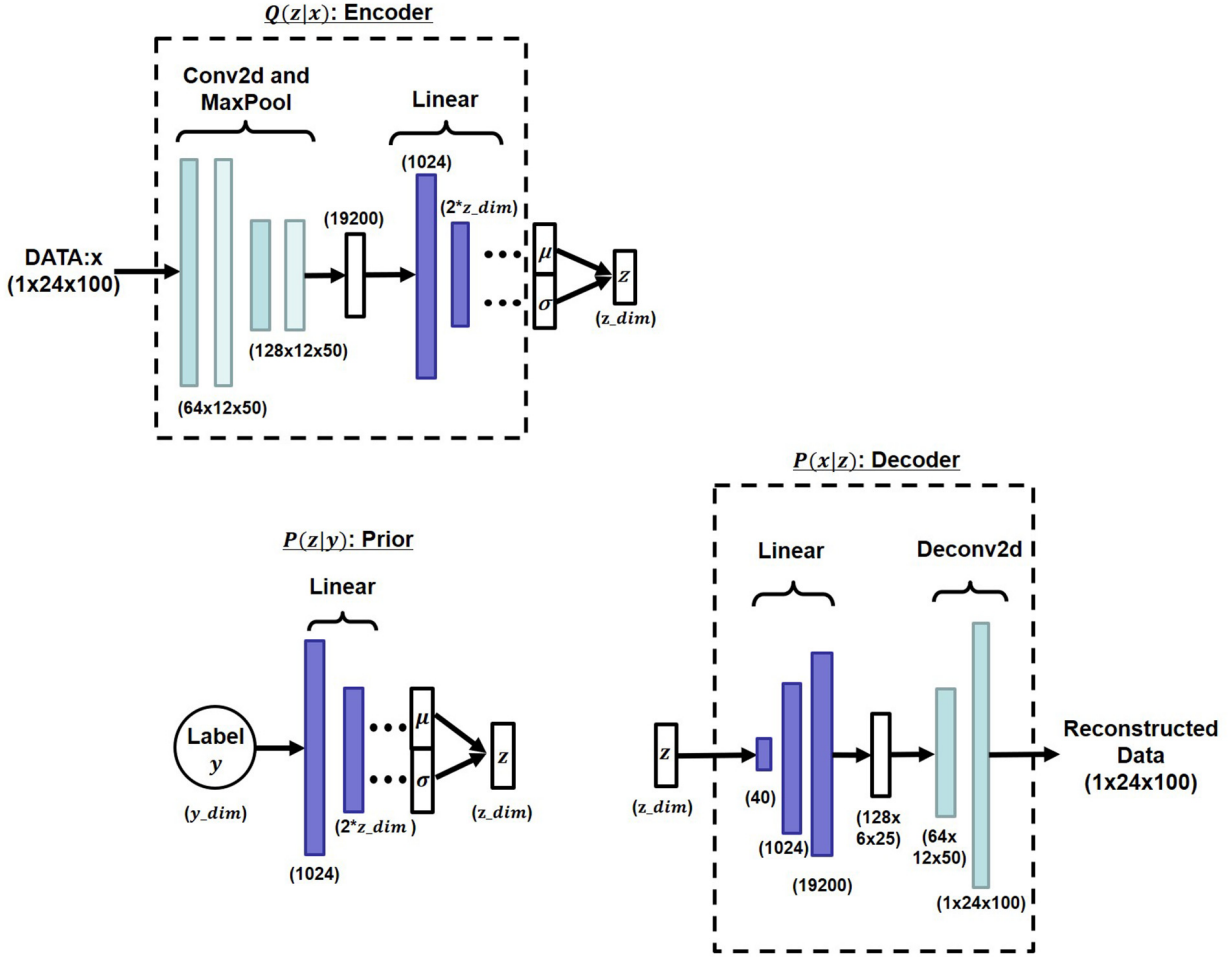
Layer structure details.

Figure 6 presents the detailed network structures of the encoder, prior, and decoder. The encoder consists of a series of two-dimensional convolutional layers followed by max pooling and a feed-forward network (FFN) to predict the mean and variance of the latent distribution. The prior network employs an FFN that maps the label to the latent space parameters. The decoder comprises an FFN followed by two-dimensional transposed convolutional layers to reconstruct the original spectrogram from the latent representation.

### 2.6 Authentication using CVAE

Biometric authentication was performed using the latent representations generated by the CVAE model. During training, heartbeat spectrograms measured from each subject were encoded into a latent space, where data points with the same subject ID gradually formed distinct clusters. Each cluster's center was calculated as the mean of the latent variables corresponding to that subject's training samples.

**Figure 7:**
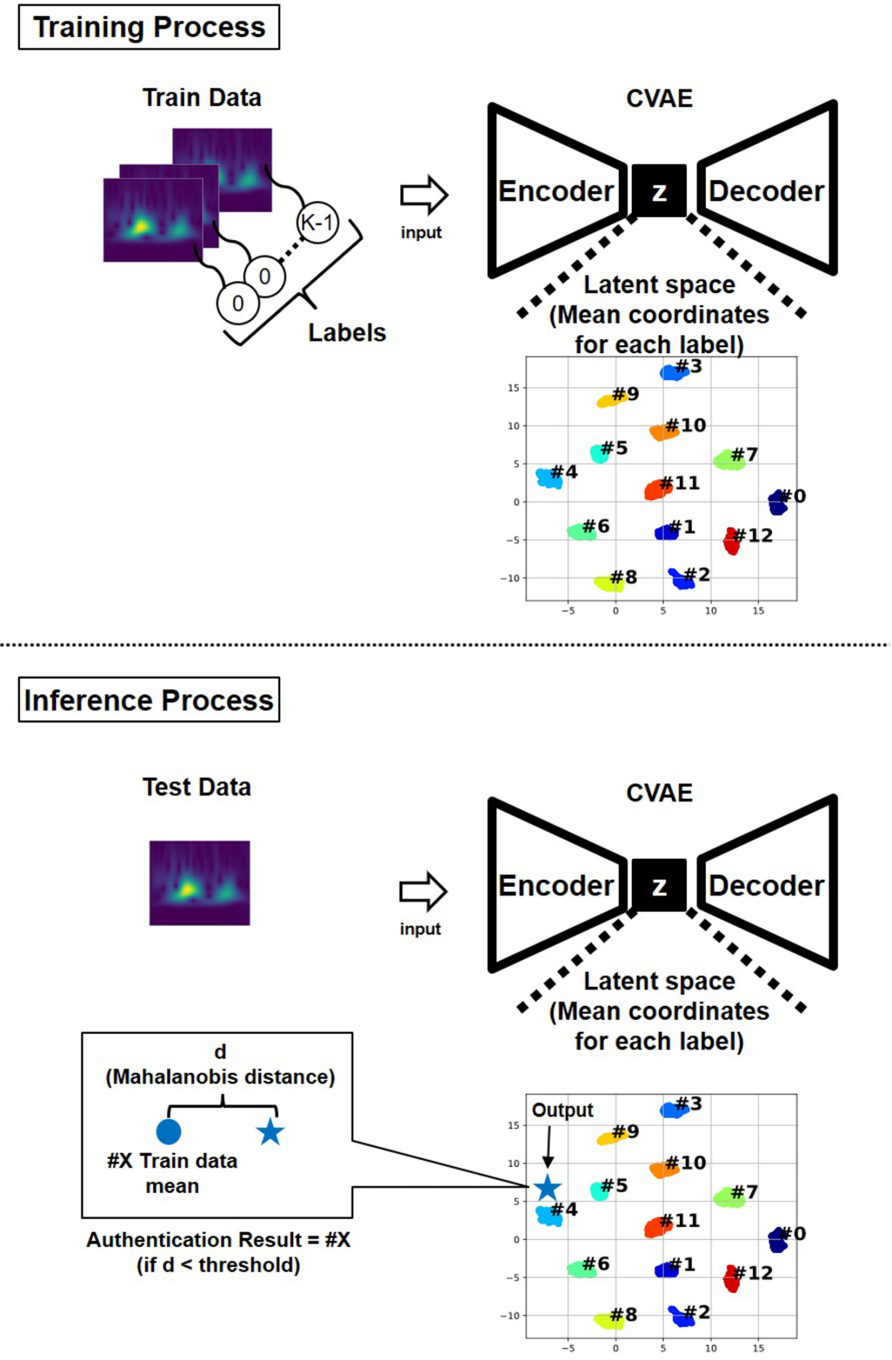
Architecture of the proposed model.

The authentication process is depicted in Figure 7. In the inference phase, test spectrograms were encoded into the latent space using the trained encoder. The distance between each encoded representation and the center of each subject's cluster was then calculated (24). In this study, the Mahalanobis distance was used. A test sample was authenticated as belonging to a subject if its Mahalanobis distance to that subject's cluster center was smaller than a predefined threshold; otherwise, it was rejected as an unregistered individual.

This distance-based authentication approach enabled robust and interpretable decision-making in the latent space structured by the CVAE model. The hyperparameters used for training the CVAE model were: batch size of 64, 300 training epochs, Adam optimizer with a learning rate of 0.00001, and a latent space dimension of 50.

## 3 Results

### 3.1 Evaluation and method

In this study, 48 s of the measured 60-s data were used for training, and the remaining 12 s were used for performance evaluation. However, the data used in these processes were not used as training data for the conformer-based heartbeat peak extraction.

First, leave-one-out cross-validation (LOOCV) was conducted to evaluate the authentication performance that proved the identity of a specific individual. In this method, the model was trained by excluding the data of one of the 13 subjects. This approach prevented data bias and enabled the assessment of the generalization performance. Of the data from the 12 subjects not excluded by LOOCV, data from one subject were used as a single label for training, whereas the data from the remaining 11 were aggregated into a single label. To balance the data volume, 1/11 of each subject's data was extracted from the remaining 11 to create the training dataset. Finally, the performance in rejecting unregistered subjects was evaluated using inference data from those excluded from LOOCV.

Subsequently, the data of all 13 subjects were trained as separate labels to evaluate the identification performance, which determines which registered individual matched the data. As the identification task involved selecting the subject most closely matched among the registered total, LOOCV was not conducted. For inference, 12 s of data not used in the training for all registered subjects were used to evaluate the performance of correctly identifying registered subjects.

During the inference process, a majority-voting method was employed using multiple consecutive heartbeat waveforms, specifically, majority voting on data from five heartbeats.

**Figure 8:**
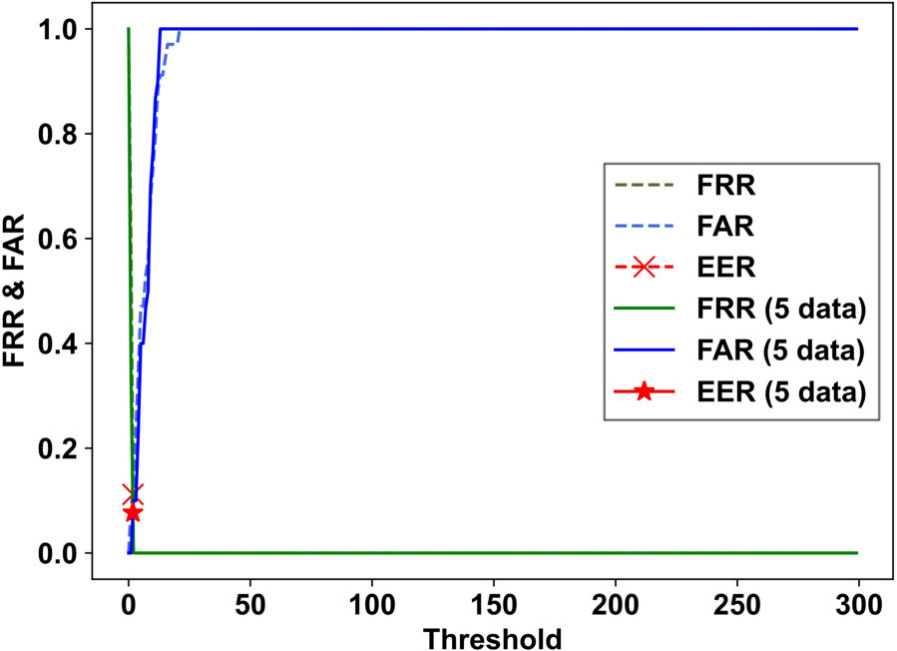
Example of the correlation between FRR and FAR with varying Mahalanobis distance thresholds in the latent space.

The results obtained were used to illustrate the correlation between the false rejection rate (FRR) and false acceptance rate (FAR) when the Mahalanobis distance threshold in the latent space was varied, as shown in Figure 8. Given that these values were correlated, they fluctuated based on the set threshold. The equal error rate (EER) was achieved when the Mahalanobis distance threshold was adjusted such that FRR and FAR were equal. This evaluation was conducted for all the subjects.

### 3.2 Metrics

The accuracy of authentication was evaluated using balanced accuracy (BAC), F1-score, and EER, whereas the accuracy of identification was evaluated using ACC.

BAC is a measure used to evaluate classifier performance on unbalanced datasets. It is the average of the true positive rate (TPR) and true negative rate (TNR), which evaluates the prediction accuracy for each class equally.

### 3.3 Experimental results

The results of calculating the average BAC, F1-score, and EER for each subject during authentication are shown in Table 1. Majority voting was performed using multiple consecutive heartbeat waveforms. Majority voting with five heartbeat datasets resulted in a BAC of 97.3%. For identification, the average ACC for each subject was calculated, with the results presented in Table 2. The majority of the votes using the five heartbeat datasets resulted in an ACC of 94.7%. These results indicate that extending the duration used for authentication and performing inferences based on multiple heartbeat signals can improve the accuracy.

**Table 1:** Evaluation scores in authentication (macro average).

Method	BAC [%]	F1-score [%]	EER [%]
Using single data	88.3	88.3	11.8
Majority voting using 5 data	**97**.**3**	**97**.**3**	**2**.**7**

The bold values indicate the highest score for each evaluation metric.

**Table 2:** Evaluation scores in identification (macro average).

Method	ACC [%]
Using single data	93.3
Majority voting using 5 data	**94**.**7**

The bold values indicate the highest score for each evaluation metric.

Additionally, Table 3 presents subject-wise evaluation results, allowing for further analysis of individual differences in performance.

**Table 3:** Evaluation scores for each subject.

Subject#	BAC [%]	F1-score [%]	EER [%]
0	100.0	100.0	0.0
1	100.0	100.0	0.0
2	93.8	93.3	5.7
3	100.0	100.0	0.0
4	100.0	100.0	0.0
5	99.5	99.5	1.0
6	95.6	95.8	4.6
7	100.0	100.0	0.0
8	83.3	83.8	17.6
9	100.0	100.0	0.0
10	92.3	92.7	6.4
11	100.0	100.0	0.0
12	100.0	100.0	0.0

To evaluate the impact of sensor position shifts and changes in heartbeat waveforms over time, two subjects were re-measured at the same location on different days. The results inferred from the re-measured data are shown in Table 4. In this data, the BAC and F1-score decreased by 4.0% and 7.2%, respectively, while the EER increased by 4.8%.

**Table 4:** Changes in evaluation scores over time.

Subject#	Measured Date	BAC [%]	F1-score [%]	EER [%]
1	2023.12.19	100.0	100.0	0.0
10	2023.12.22	92.3	92.7	6.4
	Avg.	**96** **.** **2**	**96**.**3**	**3**.**2**
1 (2nd)	2023.12.30	91.7	86.5	9.9
10 (2nd)	2024.01.12	92.8	91.7	6.1
	Avg.	**92**.**2**	**89**.**1**	**8**.**0**

The bold values represent the average results of two subjects.

## 4 Discussion

Table 5 compares the performance of previous heartbeat-based contactless authentication methods. Prior studies using microwave Doppler sensors (7) demonstrated that feature extraction through time-frequency analysis and K-nearest neighbor search could be applied for authentication. However, the accuracy of these methods was insufficient. In our previous study (8), we used a 24 GHz microwave Doppler sensor for heartbeat extraction. This method relied on AR model-based time–frequency analysis and spectrogram correlation. The sensor was fixed to the front of the chest using a belt; however, body motion noise could not be completely eliminated. Machine learning-based methods such as SVM (6, 9, 10, 13, 14), DCNN (10), and CAE (11) have also been proposed. These methods, however, face limitations such as experimental complexity (6, 14), low accuracy, small sample sizes (9, 10, 13), and restricted applicability (11).

**Table 5:** Comparison with prior works.

Study	Number of Subjects	Algorithm	Preprocessing	Authentication	Identification
Lin et al. (6)	78	SVM	Fiducial points of time series wave	BAC: 98.6% EER: 4.42%	n/a
Rissacher and Galy (7)	26	KNN	CWT	Rank-1 ACC: 19% Rank-5 ACC: 42%	n/a
Okano et al. (8)	11	RSS	AR model	ACC: 92.8% EER: 3.9%	n/a
Shi et al. (9)	4	SVM	Complexity of time series wave	n/a	ACC: 94.6%
Cao et al. (10)	4/10	CNN	STFT	n/a	ACC: 98.5%/80.7%
Huang et al. (11)	105	CAE	Fiducial features of time series wave	BAC: 96.5% EER: 3.8%	n/a
Hinatsu and Wada (13)	13	SVM	AC/DCT + MFCC	ACC: 92.3% EER: 6.1%	n/a
Kobayashi et al. (14)	6/30 (public dataset)	SVM	MFCC	n/a	ACC: 96.3%/99.4%
This work	13	CVAE	CWT	BAC: 97.3% EER: 2.7%	ACC: 94.7%

SVM, support vector machine; KNN, k-Nearest Neighbor; RSS, residual sum of squares; CNN, convolutional neural networks; CAE, convolutional autoencoder; CWT, continuous wavelet transform; AR model, autoregressive model; STFT, short-time Fourier transform; AC, autocorrelation; DCT, discrete cosine transform; MFCC, mel-frequency cepstral coefficient; ACC, accuracy; BAC, balanced accuracy; EER, equal error rates.

In contrast, the proposed method achieves high authentication and identification performance while offering enhanced usability. Unlike many previous systems, it does not require users to wear sensors or maintain constrained postures. Our system enables high-accuracy authentication without forcing users to fix a sensor to the body with a belt or remain seated in a specific position. Although our experiment was conducted in a seated posture, the non-contact approach allows natural use without burdening the user and is expected to be adaptable to other postures as well. Compared with existing methods requiring controlled experimental setups, our approach has the potential to expand the range of applicable scenarios. This flexibility, combined with high accuracy, is a key strength of the proposed system.

In terms of cost, the 60 GHz Doppler sensor used in this study offers higher frequency and accuracy than lower-frequency alternatives (e.g., 2.4 or 24 GHz), placing it in a mid-range price tier. Some previous studies have employed high-precision but specialized radar systems that may limit widespread adoption. In contrast, our method uses a commercially available radar module that enables non-contact authentication without requiring physical attachment, offering advantages in accessibility, ease of setup, and user experience.

Although the proposed method exhibited high authentication and identification accuracy, a potential limitation is the small sample size. In this study, we proposed a method for non-contact measurement of heartbeat signals using a microwave Doppler sensor and a framework for personal authentication and identification based on these signals. The method was validated using spectrograms from 13 individuals. Results showed that the proposed method achieved an average BAC of 97.3% for authentication and an average ACC of 94.7% for identification. By comparison, Huang et al. (11) reported a BAC of 96.5% and an EER of 3.8%. Our method improved the EER to 2.7%, a 28.9% relative improvement, and increased BAC by 0.8% points. ACC was comparable with the 94.6% reported by Shi et al. (9).

The re-measurement experiment, which simulated temporal variation in sensor conditions, showed a modest decline in performance (BAC −4.0%, F1-score −7.2%, EER +4.8%). This degradation appears acceptable for practical use, as the envisioned application scenario assumes a fixed device installation (e.g., in a specific room or vehicle) without repositioning after initial calibration. Under this assumption, the ability to authenticate previously enrolled users after a time interval remains sufficient for real-world deployment.

Regarding the number of subjects, we agree that evaluating generalizability with a larger and more diverse population is scientifically important. However, the intended use scenario for this system assumes deployment in relatively small communities, such as research groups, internal organizational teams, or households. In such cases, optimizing the model for a limited user base is both practical and effective. Therefore, we believe the current sample size remains meaningful from a practical perspective. Nonetheless, we plan to expand the subject pool in future work to investigate scalability and robustness further.

Although remote authentication is not the primary focus of this study, the proposed method assumes that the target user remains relatively stationary. We recognize that achieving robust authentication under light movement conditions would enhance the system's practicality. We consider this an important direction for future research aimed at improving the robustness and versatility of the approach.

Future work should not only examine the impact of increasing the number of subjects on authentication accuracy, but also develop methods that account for variations in biometric signals due to changes in posture and physical condition. Improving robustness to postural variation remains a key challenge, and algorithms capable of adapting to such changes must be established. For example, an authentication system should function reliably even when the user is leaning forward or resting against a chair. Furthermore, it is essential to maintain stable authentication performance under conditions involving light body movement. Achieving this will require advances in signal processing and machine learning algorithms to extract consistent and invariant heartbeat components across diverse measurement conditions.

## Data Availability

The raw data supporting the conclusions of this article will be made available by the authors, without undue reservation.

## References

[1] Hall JE. Guyton and Hall Textbook of Medical Physiology E-book. Philadelphia, PA: Elsevier Health Sciences (2015).

[2] Arteaga-Falconi JS Al Osman H El Saddik A. ECG authentication for mobile devices. IEEE Trans Instrum Meas. (2016) 65:591–600. 10.1109/TIM.2015.2503863

[3] Choi HS Lee B Yoon S. Biometric authentication using noisy electrocardiograms acquired by mobile sensors. IEEE Access. (2016) 4:1266–73. 10.1109/ACCESS.2016.2548519

[4] Gutta S Cheng Q. Joint feature extraction and classifier design for ECG-based biometric recognition. IEEE J Biomed Health Inform. (2016) 20:460–8. 10.1109/JBHI.2015.2402199 25680220

[5] Sun L Zhong Z Qu Z Xiong N. PerAE: an effective personalized autoencoder for ECG-based biometric in augmented reality system. IEEE J Biomed Health Inform. (2022) 26:2435–46. 10.1109/JBHI.2022.3145999 35077376

[6] Lin F Song C Zhuang Y Xu W Li C Ren K. Cardiac scan: a non-contact and continuous heart-based user authentication system. Proceedings of the 23rd Annual International Conference on Mobile Computing and Networking (2017). p. 315–28. 10.1145/3117811.3117839

[7] Rissacher D Galy D. Cardiac radar for biometric identification using nearest neighbour of continuous wavelet transform peaks. Proc. ISBA 2015; Hong Kong (2015). p. 1–6. 10.1109/ISBA.2015.7126356

[8] Okano T Izumi S Kawaguchi H Yoshimoto M. Non-contact biometric identification and authentication using microwave Doppler sensor. Proc. BioCAS 2017; Torino, Italy (2017). p. 1–4. 10.1109/BIOCAS.2017.8325160

[9] Shi K Will C Weigel R Koelpin A. Contactless person identification using cardiac radar signals. Proc. I2MTC 2018; Houston, Texas, USA (2018). p. 1–6. 10.1109/I2MTC.2018.8409645

[10] Cao P Xia W Li Y. Heart ID: human identification based on radar micro-Doppler signatures of the heart using deep learning. Remote Sens (Basel). (2019) 11:1220. 10.3390/rs11101220

[11] Huang Y Qiu M Chen L Peng Z Zhang Q Wu K. NF-heart: a near-field non-contact continuous user authentication system via ballistocardiogram. Proc ACM Interact Mob Wearable Ubiquitous Technol. (2023) 7:1–24. 10.1145/3580851

[12] Wu S Sakamoto T Oishi K Sato T Inoue K Fukuda T Person-specific heart rate estimation with ultra-wideband radar using convolutional neural networks. IEEE Access. (2019) 7:168484–94. 10.1109/ACCESS.2019.2954294

[13] Hinatsu S Wada S. Biometric authentication using chest wall displacement recorded by VHF-band loop antenna. IEEE Access. (2024) 12:196475–87. 10.1109/ACCESS.2024.3521013

[14] Kobayashi H Tanaka Y Sakamoto T. Individual identification using radar-measured respiratory and heartbeat features. IEEE Access. (2024) 12:190972–87. 10.1109/ACCESS.2024.3517668

[15] Kingma DP Rezende DJ Mohamed S Welling M. Semi-supervised learning with deep generative models. Proc. NIPS 2014; Montreal, Canada (2014). p. 3581–9

[16] Sohn K Lee H Yan X. Learning structured output representation using deep conditional generative models. Proc. NIPS 2015; Montreal, Canada (2015). p. 3483–91

[17] Mora N Cocconcelli F Matrella G Ciampolini P. Detection and analysis of heartbeats in seismocardiogram signals. Sensors. (2020) 20:1670. 10.3390/s20061670 32192162 PMC7146295

[18] Gulati A Qin J Chiu CC Parmar N Zhang Y Yu J Conformer: convolution-augmented transformer for speech recognition (2020).

[19] Cohen L. Time-Frequency Analysis. Upper saddle River, NJ: Prentice Hall (1995).

[20] Torrence C Compo GP. A practical guide to wavelet analysis. Bull Am Meteorol Soc. (1998) 79:61–78. 10.1175/1520-0477(1998)079<0061:APGTWA>2.0.CO;2

[21] Shorten C Khoshgoftaar TM. A survey on image data augmentation for deep learning. J Big Data. (2019) 6:60. 10.1186/s40537-019-0197-0 PMC8287113 34306963

[22] Zhang H Cisse M Dauphin YN Lopez-Paz D. Mixup: beyond empirical risk minimization. Proc. ICLR 2018; Vancouver, BC, Canada (2017).

[23] Kingma DP Welling M. Auto-encoding variational Bayes. Proc. ICLR 2014; Banff, AB, Canada (2013).

[24] Zhu Q Zhang R. A classification supervised auto-encoder based on predefined evenly-distributed class centroids (2019).

